# Consuming a multi-ingredient thermogenic supplement for 28 days is apparently safe in healthy adults

**DOI:** 10.3402/fnr.v59.27999

**Published:** 2015-07-22

**Authors:** Roxanne M. Vogel, Jordan M. Joy, Paul H. Falcone, Matt M. Mosman, Michael P. Kim, Jordan R. Moon

**Affiliations:** 1 MusclePharm Sports Science Institute, Denver, CO, USA; 2 Department of Human Performance, Concordia University Chicago, River Forest, IL, USA; 3 Department of Sports Exercise Science, United States Sports Academy, Daphne, AL, USA

**Keywords:** weight loss supplement, safety, clinical, hematology, nutrition, fat burner

## Abstract

**Background:**

Thermogenic (TRM) supplements are often used by people seeking to decrease body weight. Many TRM supplements are formulated with multiple ingredients purported to increase energy expenditure and maximize fat loss. However, in the past some TRM ingredients have been deemed unsafe and removed from the market. Therefore, it is important to verify the safety of multi-ingredient TRM supplements with chronic consumption.

**Objective:**

To assess the safety of daily consumption of a multi-ingredient TRM supplement over a 28-day period in healthy adults.

**Design:**

Twenty-three recreationally active adults (11M, 12F; 27.1±5.4 years, 171.6±9.6 cm, 76.8±16.1 kg, 26±5 BMI) were randomly assigned either to consume a multi-ingredient TRM supplement (SUP; *n=*9) or remain unsupplemented (CRL; *n=*14) for 28 days. Participants maintained their habitual dietary and exercise routines for the duration of the study. Fasting blood samples, resting blood pressure, and heart rate were taken before and after the supplementation period. Samples were analyzed for complete blood counts, comprehensive metabolic, and lipid panels.

**Results:**

Significant (*p*<0.05) group by time interactions were present for diastolic BP, creatinine, estimated glomerular filtration rate (eGFR), chloride, CO_2_, globulin, albumin:globulin (A/G), and high-density lipoprotein (HDL). Dependent *t*-tests conducted on significant variables revealed significant (*p<*0.05) within-group differences in SUP for diastolic BP (+6.2±5.3 mmHG), creatinine (+0.09±0.05 mg/dL), eGFR (−11.2±5.8 mL/min/1.73), globulin (−0.29±0.24 g/dL), A/G (+0.27±0.23), and HDL (−5.0±5.5 mg/dL), and in CRL for CO_2_ (−1.9±1.5 mmol/L) between time points. Each variable remained within the accepted physiological range.

**Conclusion:**

Results of the present study support the clinical safety of a multi-ingredient TRM containing caffeine, green tea extract, and cayenne powder. Although there were statistically significant (*p*<0.05) intragroup differences in SUP from pre- to postsupplementation for diastolic BP, creatinine, eGFR, globulin, A/G, and HDL, all remained within accepted physiological ranges and were not clinically significant. In sum, it appears as though daily supplementation with a multi-ingredient TRM is safe for consumption by healthy adults for a 28-day period.

Over the past decade, the prevalence of overweight and obesity in the United States has continued to rise, with approximately two-thirds of the adult population fitting into these categories (1). As the health risks associated with overweight and obesity have become apparent (2), many individuals are attempting to either control or lose weight (3). Thermogenic (TRM) supplements are often viewed as a viable option for those looking to decrease body weight or fat mass. A survey of 3,500 American adults revealed that of the respondents who had attempted to lose weight in the past 12 months, one-third had tried dietary supplements at least once to help achieve their goals (4).

Many TRM supplements are formulated with multiple ingredients purported to increase energy expenditure (EE), increase fat oxidation, or suppress appetite. Stimulants such as caffeine anhydrous and green tea extract are often combined to achieve these effects. Caffeine is a sympathetic nervous system stimulant known to increase EE and fat oxidation following acute ingestion (5, 6). Green tea extract, which contains both caffeine and catechin polyphenols, has similarly been shown to increase 24-h EE by 4% and alter substrate utilization in favor of fat oxidation (7). In long-term studies, green tea extract has also been suggested to aid weight loss and weight management following weight loss (8). Capsaicin, a bioactive compound found in hot red peppers, is another ingredient found in TRM supplements that has been shown to suppress appetite and energy intake (9) and counteract decrements in EE that are often associated with a negative energy balance diet (10). Combined, these TRM ingredients may work in a synergistic manner to potentiate weight loss or management efforts.

However, caution must also be exercised when considering TRM supplements that combine multiple active ingredients. As has been the case in the past, some of the most efficacious TRM ingredients have been deemed unsafe for consumption and subsequently removed from the market by the Food and Drug Administration. Ephedra and 1,3-dimethylamylamine (DMAA) provide two such examples (11, 12), wherein the potential benefits associated with the products were outweighed by evidence of a significant health risk posed to consumers. Indeed, a common concern associated with TRM supplements is that sufficient scientific data have not been collected on them prior to release, with marketing efforts taking priority over evaluation of safety and efficacy (13). Furthermore, in a recent review of common dietary supplements for weight loss, Saper et al. (14) concluded that none of the more than 20 individual ingredients studied met the criteria for recommendation in clinical practice. To meet the criteria for recommendation, the product needed to exhibit ‘strong evidence for the presence of quality, safety, and efficacy’ based on the existing literature (14). Such conclusions highlight the importance of randomized, controlled clinical trials that examine the safety and efficacy of over the counter weight loss products for health care providers making recommendations to their patients.

It is therefore prudent to verify the safety and potential side effects of TRM supplements currently on the market. The purpose of the present study was to assess the clinical safety of daily consumption of a multi-ingredient TRM supplement containing caffeine, green tea extract, and capsaicin-containing cayenne fruit powder over a period of 28 days in healthy men and women. It was hypothesized that daily supplementation would not produce abnormal changes in hematological or metabolic safety markers or resting vital signs.

## Methods

### Experimental design

**Fig. 1:**
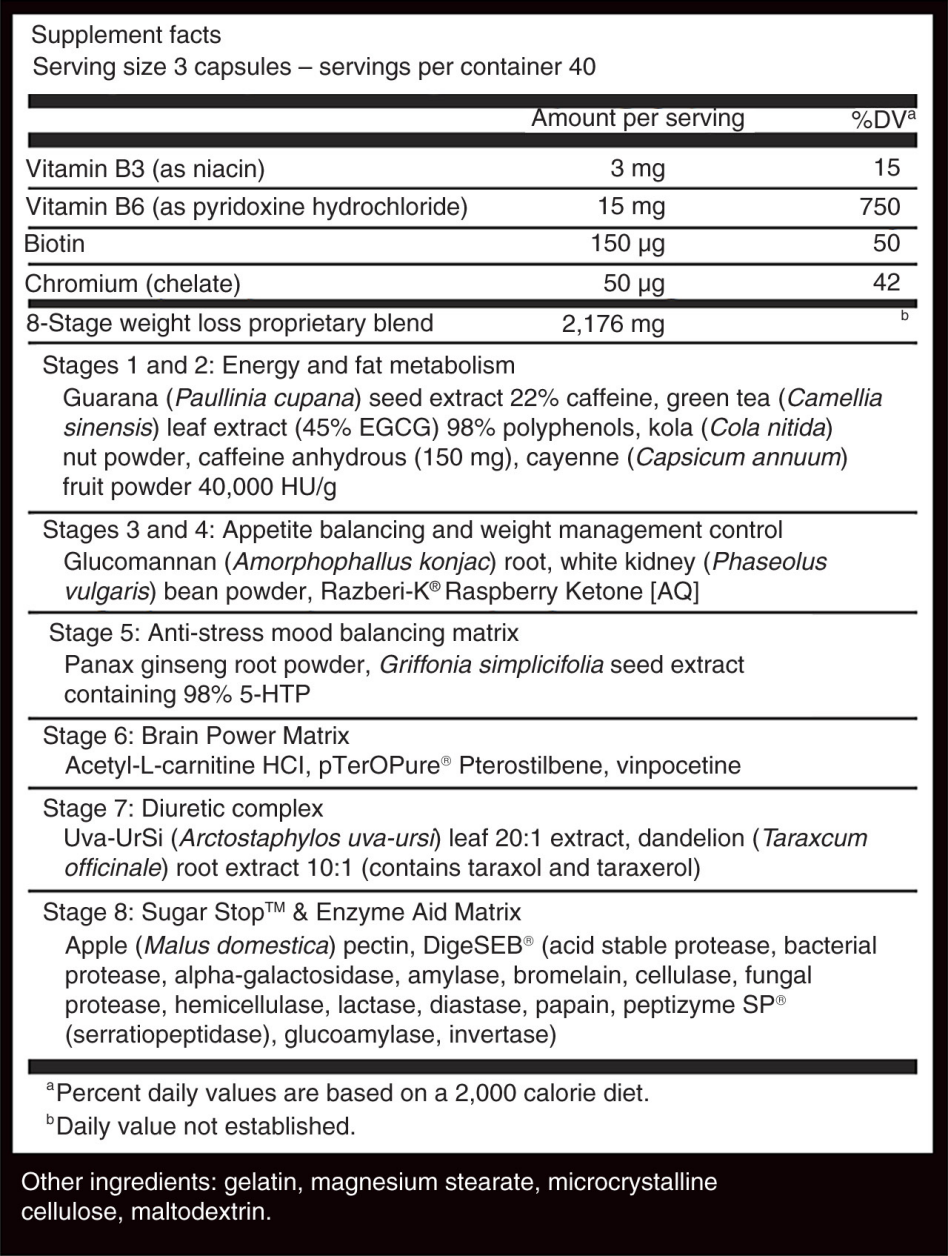
Supplement facts.

In a randomized design, 23 subjects (12 female, 11 male) were randomly assigned to control (CRL, *n*=14; 26.5±5.9 years, 173.6±9.1 cm, 77.0±17.8 kg) or supplement (SUP, *n*=9; 28.0±4.7 years, 168.5±10.0 cm, 76.6±14.2 kg) groups via random number generation by the investigators and asked either to remain unsupplemented or consume two servings, respectively, of a commercially available TRM (MuslcePharm Shred Matrix™, MusclePharm Corp., Denver, CO) every day for 28 days. Supplement facts are listed in Fig. 1. The quality and integrity of the finished product was assessed and verified by an independent, third-party analytical laboratory (Eurofins Scientific Inc., Petaluma, CA, USA). Subjects in SUP were instructed to consume one serving (three capsules) of the TRM in the morning 30–45 min prior to breakfast with at least 8 oz water, as well as one serving (three capsules) 30–45 min prior to lunch with at least 8 oz water. Compliance was monitored using supplement consumption logs, as well as by quantifying the number of capsules in the supplement containers before and after the supplementation period. Participants completed the study with an average supplementation compliance of 96.5%. Blood draws were taken prior to and following the supplementation period. Approval for the human subject protocol was obtained from a registered IRB, and subjects provided written informed consent prior to their participation in the study.

### Participants

Twenty-three recreationally active adults (12 female: 28.4±5.3 years, 164.1±4.8 cm, 71.2±17.9 kg, BMI 26.4±6.4 kg·m^−2^; 11 male: 25.6±5.5 years, 179.7±6.0 cm, 83.0±11.8 kg, BMI 25.6±3.0 kg·m^−2^) participated in the present study. Recreationally active was defined as habitually participating in moderate to vigorous physical activity on three or more days a week for a duration of 30 min or more. Subjects were required to be non-smokers, free of any disease or disorder which may have produced confounding effects, and have abstained from taking any other TRM or weight-loss supplements for 1 month prior to the beginning of the study. Eligibility was determined upon evaluation of preparticipation health history, exercise, and supplementation screening questionnaires. Subjects were instructed to maintain their habitual dietary and exercise routines and not to take any additional supplements during their participation in the study. Furthermore, participants were cautioned against changing any aspect of their current diet and exercise regimen in attempts to decrease body weight.

### Measurements

All measurements were taken prior to and following the 28-day supplementation period. Upon arrival at the laboratory, height and weight as well as resting blood pressure and heart rate were recorded using a high-capacity column scale with built-in stadiometer (SECA 703, SECA, Hamburg, Germany), and an automated digital sphygmomanometer (Omron BP786, Omron Corporation, Kyoto, Japan), respectively. Subjects then submitted a blood sample in the morning following an overnight fast to control for diurnal fluctuations. Blood draws were performed by a trained phlebotomist via venipuncture, and samples were analyzed for comprehensive metabolic panels, complete blood counts, and lipid profiles by an external laboratory (Laboratory Corporation of America, Denver, CO). Variables recorded from blood analysis consisted of white blood cell count (WBC), red blood cell count (RBC), hemoglobin, hematocrit, mean corpuscular volume (MCV), mean corpuscular hemoglobin (MCH), mean corpuscular hemoglobin concentration (MCHC), red blood cell distribution width (RDW), platelets (absolute), neutrophils (percent and absolute), lymphocytes (percent and absolute), monocytes (percent and absolute), eosinophils (percent and absolute), basophils (percent and absolute), serum glucose, blood urea nitrogen (BUN), creatinine, estimated glomerular filtration rate (eGFR), BUN:creatinine, sodium, potassium, chloride, carbon dioxide (CO_2_), calcium, protein, albumin, globulin, albumin:globulin (A/G) ratio, bilirubin, alkaline phosphatase, aspartate aminotransferase (AST), alanine aminotransferase (ALT), total cholesterol, triglycerides, high-density lipoprotein (HDL) cholesterol, and low density lipoprotein (LDL) cholesterol. Intertest reliability results from 12 men and women measured up to 1 week apart at the aforementioned laboratory resulted in no significant differences from day-to-day (*p*>0.05) and an average intertest Coefficient of Variation of <6.9% for all tests.

### Statistical analyses

Data were analyzed using a 2 [CRL vs. SUP]×2 [pre vs. post] repeated measures ANCOVA model for all group, time, and group by time interactions with the pre-value as the covariate. Alpha was accepted at *p*<0.05 level of significance. Variables demonstrating a significant (*p*<0.05) group by time interaction were then analyzed for within-group differences using dependent *t*-tests, in addition to independent *t*-tests conducted on the mean delta values to examine between-group differences in change over time. Shapiro–Wilk tests were used to determine normality of the data. The minimal difference (MD) needed to be considered real was determined using the method previously described by Weir (15). Data were analyzed using Statistica v. 10 software (StatSoft, Inc., Tulsa, OK, USA).

## Results

Significant (*p*<0.05) group by time interactions were observed for diastolic blood pressure, creatinine, eGFR, chloride, CO_2_, globulin, A/G ratio, and HDL cholesterol. Dependent *t*-tests revealed within-group differences between time points in SUP for diastolic blood pressure (+6.2±5.3 mmHG, *p*=0.008), creatinine (+0.09±0.05 mg/dL, *p*=0.001), eGFR (−11.2±5.8 mL/min/1.73, *p*<0.001), globulin (−0.29±0.24 g/dL, *p*=0.006), A/G ratio (+0.27±0.23, *p*=0.008), and HDL cholesterol (−5.0±5.5 mg/dL, *p*=0.027). In CRL, dependent *t*-tests revealed intragroup differences for CO_2_ from pre- to postsupplementation (−1.9±1.5 mmol/L, *p*=0.001). For chloride, neither group reached significance (*p*>0.05) for dependent *t*-tests. Each of the aforementioned variables remained within accepted physiological reference ranges and were normally distributed (*p*>0.05) at both time points. Body weight was not significantly different either within or between groups from pre- to posttesting (SUP: 76.6±14.2 kg pre vs 76.2±14.6 kg post; CRL: 77.0±17.8 kg pre vs 76.9±17.2 kg post). No other variables had significant group by time interactions. Data are presented as means±SD in Table 1.

**Table 1:** Means±SD for variables with significant (*p*<0.05) group×time interactions

Variable	Group	Pre	Post	Δ	Reference interval
Resting diastolic blood pressure (mmHg)	CRL	74.1±12.7	73.4±9.2	−0.75±7.8	<80
	SUP	71.7±7.5	77.9±3.9^a^	6.2±5.3^b^	
Creatinine (mg/dL)	CRL	0.98±0.13	0.97±0.15	−0.01±0.06	0.57–1.27
	SUP	0.88±0.17	0.97±0.17^a^	0.09±0.05^b^	
eGFR (mL/min/1.73)	CRL	93.8±12.0	95.4±16.0	1.6±6.9	>59
	SUP	104.2±17.6	93.0±14.9^a^	−11.2±5.8^b^	
Chloride (mmol/L)	CRL	101.7±2.0	102.3±2.2	0.6±2.2	97–108
	SUP	100.8±2.2	100.0±1.9^a^	−0.8±1.5	
Carbon dioxide (mmol/L)	CRL	22.1±1.9	20.2±2.0	−1.9±1.5	18–28
	SUP	21.2±2.6	22.7±2.4^a^	1.4±2.6^b^	
Globulin (g/dL)	CRL	2.63±0.30	2.59±0.34	−0.04±0.11	1.5–4.5
	SUP	2.66±0.24	2.37±0.22^a^	−0.29±0.24^b^	
A/G ratio	CRL	1.71±0.23	1.74±0.28	0.03±0.11	1.1–2.5
	SUP	1.67±0.17	1.93±0.21^a^	0.27±0.23^b^	
HDL (mg/dL)	CRL	61.3±13.1	63.0±11.9	1.7±3.8	>39
	SUP	60.0±12.2	55.0±11.8^a^	−5.0±5.5^b^	

CRL=control (*n*=14); SUP=supplement (*n*=9); all data are reported as means±SD.

a Significantly different from CRL, ANCOVA (*p*<0.05).

b Significantly different from corresponding CRL delta, *t*-test (*p*<0.05).

## Discussion

The results of the present study support the hypothesis that daily TRM supplementation does not appear to cause abnormal changes in hematological and clinical chemistry or metabolic safety markers or resting vital signs in healthy adult subjects. While significant group by time interactions (*p*<0.05) were observed for diastolic blood pressure, creatinine, eGFR, chloride, CO_2_, globulin,A/G ratio, and HDL cholesterol, each of these variables remained well within the accepted physiological range and are not clinically significant. In addition, dependent *t*-tests did not reach significance (*p*>0.05) for intragroup differences in SUP for chloride or CO_2_.

Variables that were significantly different at the group level were further evaluated at the individual level to determine clinical significance. Analysis of clinical significance at the individual level was conducted using the MD statistic, which calculates the magnitude of the intertest difference (between baseline and postsupplementation) needed to be exceeded in order for a single measurement to be considered real, as described by Weir (15). If a subject exceeded the MD, the change was considered a *true* change. Clinical significance at the individual level was reached when a score that exceeded the MD crossed the upper or lower limits of the accepted physiological range for each variable. In SUP, this occurred with CO_2_ in one subject whose values increased from pre- to posttesting, going from outside the reference range to within range. Also, for HDL, one subject in SUP moved from within range to out of range, with values decreasing over time. However, results from one individual are not enough evidence to indicate a change due to supplementation. Also worth noting is the fact that one subject from CRL reached clinical significance for CO_2_ with values increasing pre- to postsupplementation, moving outside the reference range. In addition, two CRL subjects exceeded the MD for diastolic blood pressure, decreasing over time yet remaining outside the clinical reference range at both time points. No individuals crossed the upper or lower acceptable limit for creatinine, chloride, globulin, or A/G ratio. Collectively, individual analyses support the present hypothesis and also support the notion of intrasubject diurnal variability. Such results suggest a natural variation in these clinical markers that is not necessarily attributable to supplementation.

The present findings agree with previous literature. Roberts et al. (16) examined the safety of a TRM beverage over an identical period of 28 days in healthy males and females and found it did not alter blood and clinical safety markers, nor did it have an effect on hemodynamics following daily supplementation. Belza et al. (17) concluded that a multi-ingredient TRM containing capsaicin, green tea extract, tyrosine, caffeine, and calcium had a good safety profile, was well tolerated, and showed no difference in hemodynamic response when compared to placebo after an 8-week supplementation period. Interestingly, a multi-ingredient TRM containing DMAA and caffeine was also found to be safe following 14 days of ingestion, with no significant effects from pre- to postsupplementation on hemodynamics or blood samples analyzed for complete blood counts, comprehensive metabolic panels, and lipid panels (18). However, the findings may have been limited by the small subject number (*n*=6) and short duration of the study.

The present study adds to the existing literature, suggesting that a multi-ingredient TRM containing caffeine, green tea extract, and cayenne powder is safe for daily consumption up to 28 days, at least with regard to hematological and metabolic clinical markers of safety and hemodynamics. Again, while none of the significant variables left the accepted physiological range, the possibility that they might indicate the beginning of adverse trends cannot be ruled out. Future studies should examine the effects of supplementation for longer than 28 days among more subjects, especially given the fact that statistically significant interactions did take place over time in the present study. In addition, the present study involved only modestly overweight (average BMI of 26±5 kg·m^−2^) individuals, not moderately overweight and/or obese individuals, which may be more representative of the population to which this product is likely to appeal. Perhaps another limitation is the age of the participants (27.1±5.4 years), which may make the present findings less applicable for older populations. Similar studies involving either moderately overweight or obese and older subjects are therefore warranted. Follow-up investigations may also examine the supplement's effect on EE, substrate utilization, and efficacy as a weight loss aid.

## Conclusion

Daily supplementation with a multi-ingredient TRM does not appear to have adverse effects on markers of clinical safety or resting vital signs among healthy, recreationally active adults for a period of 28 days. The results suggest that chronic consumption of the multi-ingredient TRM supplement used in the present study is safe in healthy, young individuals for up to 1 month. Future investigations may seek to expand on the current findings by utilizing a longer supplementation period and a larger sample size to verify long-term safety.

## Supplementary Material

Consuming a multi-ingredient thermogenic supplement for 28 days is apparently safe in healthy adults
